# Clinical characteristics of fatigued Parkinson’s patients and the response to dopaminergic treatment

**DOI:** 10.1186/s40035-016-0056-2

**Published:** 2016-05-10

**Authors:** Rao Fu, Xiao-Guang Luo, Yan Ren, Zhi-Yi He, Hong Lv

**Affiliations:** Neurology Department, Outpatient of Parkinson’s Disease, First Affiliated Hospital of China Medical University, 155# Nanjing bei street, Heping District Shenyang, 110001 P R China

**Keywords:** Parkinson’s disease, Fatigue, Sleep disorder, Depression, Dopaminergic drugs

## Abstract

**Background:**

Fatigue, which is commonly observed in Parkinson’s disease (PD), can greatly reduce quality of life and is difficult to treat. We here aimed to investigate the prevalence and characteristics of fatigue among PD patients and to explore an effective strategy to treat PD fatigue.

**Method:**

This was an observational cross-sectional study conducted in northeastern China. We examined fatigue in 222 PD patients from northeastern China using the Parkinson Fatigue Scale-16 (PFS-16). The disease severity, depression, sleep and cognitive functioning were assessed with the Hoehn & Yahr staging (H-Y stage), Unified Parkinson’s Disease Rating Scale (UPDRS), Hamilton Depression Scale (HAMD), Parkinson’s Disease Sleep Scale (PDSS) and Montreal Cognitive Assessment (MoCA) by interview.

**Results:**

The frequency of fatigue in PD patients was 59.46 %. Fatigued patients had longer disease durations and greater disease severity than nonfatigued patients. Additionally, fatigued PD patients scored significantly higher for all motor symptoms, except for tremor, and had more serious depressive symptoms and sleep disturbances than nonfatigued PD patients did. The sleep disturbance severity was an independent factor for fatigue. Furthermore, 43.04 % of fatigued patients taking dopaminergic drugs had fatigue remission. Depression severity was identified as an independent factor for dopaminergic drug non-responsive fatigue.

**Conclusions:**

PD patients with severe sleep disturbances tend to suffer from fatigue. Levodopa improved fatigue only in PD patients with mild depression or no depression, implying that dopaminergic medication is required, but not sufficient, for fatigue suppression in PD patients with moderate or severe depression. Thus, restoring serotonergic neurotransmission as a combination therapy may offer a better strategy for the treatment of fatigue in these patients.

## Background

Fatigue is one of the most common non-motor symptoms in Parkinson disease (PD). Currently, there is no universally accepted definition of fatigue in PD. PD patients complaining about fatigue describe it as a sensation of tiredness, lack of energy, or exhaustion. These states are experienced as abnormally severe. It is often unpredictable in its onset and duration, often attributable to activity levels [1]. According to a recent study in PD, fatigue is one of the most bothersome problems [2], which has been confirmed as the most disabling symptom by 15–33 % of PD patients [3, 4]. Despite its high prevalence and negative impact on life quality, fatigue in PD remains an under-recognized problem in routine clinical practice [5].

Most of the published investigations have focused on prevalence and clinical characteristics of PD related fatigue, and found the close relationship between fatigue, sleep disturbance, depression and anxiety [6–8]. However few of them explored the prevalence and profiles of dopaminergic-responsive fatigue in fatigued PD patients on dopaminergic treatment. Previous studies have shown that dopamine is involved in the development of exercise-induced fatigue [9, 10]. Decreased dopamine levels were detected in the brain using a rat model of exercise-induced fatigue [11], indicating that the shortage of dopamine contributed to the occurrence of fatigue. This is consistent with the ELLDOPA Trial [12], which noted improved fatigue with levodopa treatment, even though the benefit was minimal. However, a recent meta-analysis of the intervention for fatigue in PD concluded [13] that currently there is insufficient evidence to support the effective treatment of fatigue in PD with any drug. Clinically, we do have some patients whose fatigue can be relieved with levodopa treatment, in our personal experience. Therefore, in the present study, we specifically examined fatigue and fatigue-related clinical features in patients with PD to determine the clinical characteristics of fatigue in PD patients. More importantly, we aimed to clarify the profile of dopaminergic-responsive fatigue and its associated factors.

## Methods

### Subjects and demographic data

This was an observational cross-sectional study. Two hundred and twenty-two PD patients, diagnosed according to the UK PD Brain Bank criteria [14], were recruited between December 2012 and April 2014 from the outpatient Department of Neurology of the First Affiliated Hospital of China Medical University (Shenyang, China). Patients with other forms of parkinsonism were excluded. Patients with dementia or fatigue related to a disease other than PD were excluded (e.g., peripheral circulatory disorders, such as ischemic heart disease and left ventricular failure, or acute and chronic inflammatory demyelinating polyneuropathies, such as Guillain-Barre syndrome). All patients enrolled in the study gave written informed consent. The Ethics Review Board of First Affiliated Hospital of China Medical University (Shenyang, China) approved the protocol of the study. Demographic data were collected for all PD patients. The total amount of dopaminergic medication was expressed as the levodopa equivalent daily dosage (LEDD), which was determined using methods previously reported in a systematic review of LEDD [15].

### Assessments

Disease severity was evaluated during the on phase using the Unified Parkinson’s Disease Rating Scale (UPDRS) and Hoehn & Yahr staging (H-Y stage) [16]. As previously described [17], the following sub-scores obtained from specific items of UPDRS III were calculated as following: tremor (items 20 and 21), rigidity (item 22), bradykinesia (items 23–26 and 31) and posture/gait (items 27–30).

Fatigue was assessed using the Parkinson Fatigue Scale-16 (PFS-16). The PFS-16 is a 16-item self-report scale that is specifically designed to assess the physical aspects of fatigue in PD patients [1]. The item response options range from 1 (“strongly disagree”) to 5 (“strongly agree”). The total PFS-16 score ranges from 1 to 5 and is obtained by dividing the sum of all item scores by 16 [18]. A cut-off score of 3.3 was used to indicate the presence of fatigue [1]. Subjects with a PFS-16 score <3.3 were classified as nonfatigued, and those scoring ≥3.3 were classified as fatigued.

The Montreal Cognitive Assessment (MoCA) Beijing version was used to evaluate cognitive functioning. Because depression and sleep disturbances have been reported to affect fatigue, we revised our study protocol during the study. The 17-item Hamilton Depression Scale [19] (HAMD) and Parkinson’s Disease Sleep Scale (PDSS) [20] were administered to 177 of the 222 patients to assess depression and sleep disturbance, respectively. In the 17-item HAMD, a higher score indicates severe depression, and depression was classified as follows: no depression (0–7); mild depression (8–16); moderate depression (17–23); and severe depression (≥24) [21]. The range of PDSS score was 0–150, and a lower score indicated a higher number of sleep disturbances.

### Statistical analysis

All continuous data are presented as the mean ± standard deviation (SD). Categorical variables were compared with chi-square tests; the Mann-Whitney *U* test was used for continuous variables. Correlations were assessed using Spearman rank order correlation coefficients. The odds ratios were calculated using logistic regression and chi-square tests. All data analyses were performed using SPSS software. A value of *P* < 0.05 was considered statistically significant.

## Results

### Comparison of demography and clinical profiles between the fatigued and nonfatigued groups

According to the suggested cut-off score for the PFS-16 (≥3.3), fatigue was present in 132 (59.46 %) patients. Comparisons of the demographic and clinical characteristics between the fatigued and nonfatigued groups are listed in Table 1. The mean disease duration in the nonfatigued group was significantly shorter than that in the fatigued group (3.75 ± 3.26 years vs. 4.61 ± 2.99 years, *p* = 0.046). There were no significant differences between the two groups in other clinical characteristics, including sex, age, onset age and education.

**Table 1:** Demographic and clinical characteristics of fatigued vs.nonfatigued subjects

Characteristic	Nonfatigued	Fatigued	*P* -value
Male (*n*= 222)	54 (60.00 %)	64(48.48 %)	0.101
Age, y	61.63 ± 9.49	62.50 ± 10.21	0.524
Onset age, y (*n*= 222)	57.88 ± 9.77	57.89 ± 10.51	0.992
Duration of disease, y (*n*= 222)	3.75 ± 3.26	4.61 ± 2.99	0.046*
Education, y (*n*= 222)	10.61 ± 3.12	9.80 ± 3.69	0.09
LEDD (mg/day) (*n*= 222)	211.19 ± 263.18	280.24 ± 304.04	0.081
H-Y stage (*n*= 222)	1.73 ± 0.78	2.00 ± 0.76	0.009*
UPDRS total score (*n*= 222)	39.43 ± 20.26	52.39 ± 20.19	<0.001*
UPDRS part I (*n*= 222)	2.93 ± 1.93	4.19 ± 2.26	<0.001*
UPDRS part II (*n*= 222)	9.88 ± 4.80	12.57 ± 5.72	<0.001*
UPDRS part III (*n*= 222)	25.76 ± 15.37	33.53 ± 14.66	<0.001*
Postural/gait (sum of UPDRS item 27–30)	3.28 ± 2.80	4.80 ± 3.07	<0.001*
Bradykinesia (sum of UPDRS item 23–26,31)	12.30 ± 7.98	15.9 ± 7.35	0.001*
Rigidity (UPDRS item 22)	4.87 ± 4.23	6.45 ± 4.36	0.008*
Tremor (sum of UPDRS item20 and item21)	3.36 ± 3.26	3.78 ± 3.88	0.391
UPDRS IV (*n*= 222)	0.86 ± 1.65	1.70 ± 2.57	0.007*
MoCA (*n*= 222)	22.87 ± 4.59	21.85 ± 4.27	0.066
HAMD (*n*= 177)	7.26 ± 5.47	9.32 ± 5.17	0.006*
PDSS (*n*= 177)	119.10 ± 23.65	98.82 ± 25.74	<0.001*

Data are expressed as numbers, with percentages in parentheses, or as mean ± SD. *Significant difference. *H*-*Y stage* Hoehn & Yahr staging, *LEDD* levodopa equivalent daily dosage, *UPDRS* Unified Parkinson’ Disease Rating Scale, *MoCA* Montreal Cognitive Assessment, *HAMD* Hamilton Depression Scale score, *PDSS* Parkinson’s Disease Sleep Scale

Disease severity, as determined by H-Y stage and the total UPDRS score and all subscales, was more serious in the fatigued group than in the nonfatigued group (Table 1). Further analysis of the individual motor symptoms revealed that except for tremor, most motor symptoms, including rigidity, bradykinesia and posture/gait, were more serious (higher scores) in the fatigued group than in the nonfatigued group (Table 1). Similarly, the fatigued group had significantly worse scores for depressive symptoms and sleep disturbance than the nonfatigued group had (Table 1).

The LEDD and MoCA scores tended to be higher in the fatigued group than in the nonfatigued group, but this was not significant.

### Independent risk factors related to fatigue

As shown in Table 2, the univariate logistic regression revealed that longer disease duration, a greater disease severity (H-Y stage, total UPDRS score and all subscales), depression and sleep disturbances were associated with fatigue (*p*< 0.05). Other clinical variables, including gender, age onset age, education, LEDD and MoCA score were not significantly associated with fatigue.

**Table 2:** Logistic regression modal of the association between fatigue and clinical characteristics in PD patients

Variable	Univariate analysis		Multivariate analysis	
	odds ratio	*P* -value	odds ratio	*P* -value
Female sex (*n*= 222)	1.594 (0.926–2.742)	0.092		
Age, y (*n*= 222)	1.009 (0.982–1.037)	0.522		
Onset age, y (*n*= 222)	1.000 (0.974–1.027)	0.992		
Duration of disease, y (*n*= 222)	1.100 (1.000–1.208)	0.049*		
Education, y (*n*= 222)	0.935 (0.864–1.011)	0.091		
LEDD (mg/day) (*n*= 222)	1.001 (1.000–1.002)	0.083		
H-Y stage (*n*= 222)	1.578 (1.102–2.258)	0.013*		
UPDRS total score (*n*= 222)	1.033 (1.018–1.048)	<0.001*		
UPDRS part I (*n*= 222)	1.333 (1.159–1.533)	<0.001*		
UPDRS part II (*n*= 222)	1.122 (1.059–1.189)	<0.001*		
UPDRS part III (*n*= 222)	1.036 (1.016–1.056)	<0.001*		
UPDRS part IV (*n*= 222)	1.210 (1.049–1.396)	0.009*		
HAMD score (*n*= 177)	1.079 (1.016–1.146)	0.013*		
PDSS (*n*= 177)	0.967 (0.954–0.981)	<0.001*	0.974 (0.959–0.989)	0.001
MoCA score (*n*= 222)	0.948 (0.890–1.009)	0.094		

Figures in parentheses indicate 95 % confidence intervals. *Significant difference. *H*-*Y* stage Hoehn & Yahr staging, *LEDD* levodopa equivalent daily dosage, *UPDRS* Unified Parkinson’ Disease Rating Scale, *MoCA* Montreal Cognitive Assessment, *HAMD* Hamilton Depression Scale score, *PDSS* Parkinson’s Disease Sleep Scale

We further explored the independent risk factors of fatigue in PD patients using multivariate logistic regression. Only sleep disturbance (odds ratio = 0.974, 95%CI 0.959–0.989, *P* = 0.001) remained an independent risk factor for fatigue (Table 2). Disease duration, disease severity (H-Y stage, total UPDRS score and all subscales) and depression were not associated with fatigue (Table 2).

### Comparison of clinical variables between the dopaminergic responsive fatigue and dopaminergic non-responsive fatigue groups

To further explore the clinical profiles of dopaminergic drug responsive fatigue, we examined the data of 79 patients in the fatigued group with complaints of fatigue and who were treated with dopaminergic drugs. We defined the fatigue amelioration rate between 0 % and 100 %, and assigned fatigue remission rate into 4 level (level 0: no remission, remission rate <25 %; level 1: mild remission, remission rate between 25 % and 50 %; level 2: moderate remission, remission rate between 50 % and 75 %; level 3: obvious remission, remission rate over 75 %) with a lower level indicating less responsiveness to dopaminergic medication. According to their response to dopaminergic drugs the 79 patients were assigned to either the dopaminergic drug responsive fatigued (DDRF) group (fatigue amelioration rate > level 0) or the dopaminergic drug non-responsive fatigued (DDNRF) group (fatigue amelioration rate = level 0).

The demographic and clinical profiles were compared between the DDRF and DDRNF groups; the results of these comparisons are shown in Table 3. There were 34 (43.04 %) patients in the DDRF group and 45 (56.96 %) patients in the DDNRF group. Onset age was significantly higher in the DDNRF group than in the DDRF group, and significantly more severe depression was found in the DDNRF group. There were no significant differences between the two groups in age, sex, disease duration, education, disease severity (H-Y stage, UPDRS total score and all subscores), individual motor symptoms, sleep disturbance or cognitive functioning (Table 3).

**Table 3:** Comparison between dopaminergic drug responsive fatigue (DDRF) and dopaminergic drug non-responsive fatigue (DDNRF)

Variable	DDRF	DDNRF	*p*
Male (*n*= 79)	13 (38.24 %)	24 (53.33 %)	0.354
Age, y (*n*= 79)	59.59 ± 8.89	63.64 ± 11.80	0.074
Onset age, y (*n*= 79)	54.24 ± 8.95	58.82 ± 11.93	0.040*
Duration of disease (*n*= 79)	5.35 ± 3.02	4.82 ± 3.24	0.276
Education, y (*n*= 79)	10.03 ± 2.93	9.36 ± 4.23	0.681
LEDD (mg/day) (*n*= 79)	473.38 ± 281.42	438.26 ± 271.25	0.804
H-Ystage (*n*= 79)	2.15 ± 0.82	2.04 ± 0.74	0.526
UPDRS total (*n*= 79)	55.94 ± 21.46	52.91 ± 20.22	0.674
UPDRS I (*n*= 79)	4.06 ± 2.15	3.87 ± 2.61	0.506
UPDRS II (*n*= 79)	13.00 ± 5.65	13.59 ± 6.36	0.67
UPDRS III (*n*= 79)	36.76 ± 15.50	33.63 ± 14.67	0.362
Gait (*n*= 79)	5.12 ± 3.15	5.26 ± 3.41	0.843
Tremor (*n*= 79)	4.32 ± 4.20	320 ± 3.89	0.19
Rigidity (*n*= 79)	7.56 ± 4.59	6.20 ± 4.19	0.198
Bradykinesia (*n*= 79)	16.94 ± 7.50	16.38 ± 7.47	0.741
UPDRS IV (*n*= 79)	2.11 ± 2.77	1.82 ± 2.70	0.541
HAMD (*n*= 70)	7.66 ± 4.51 (*N*= 32)	10.66 ± 4.70 (*N*= 38)	0.006*
PDSS (*n*= 70)	107.81 ± 25.33 (*N*= 32)	97.26 ± 25.98 (*N*= 38)	0.073
MoCA (*n*= 79)	22.47 ± 4.14	21.42 ± 4.59	0.353

Data are expressed as numbers, with percentages in parentheses, or as mean ± SD. *Significant difference. *DDRF* dopaminergic drug responsive fatigue, *DDNRF* dopaminergic drug non-responsive fatigueH-Y stage: Hoehn & Yahr staging, *LEDD* levodopa equivalent daily dosage, *UPDRS* Unified Parkinson’ Disease Rating Scale, *MoCA* Montreal Cognitive Assessment, *HAMD* Hamilton Depression Scale score, *PDSS* Parkinson’s Disease Sleep Scale

### Depression severity is an independent risk factor for dopaminergic drug non-responsive fatigue

As shown in Table 4, the Spearman rank order correlation coefficients revealed significant correlations between fatigue remission rate and age, onset age, and depression severity. Other factors including sex, education, LEDD, disease severity (H-Y stage, UPDRS total score and all subscales) and cognitive functioning were not associated with the fatigue remission rate.

**Table 4:** Correlations between fatigue remission rate and clinical characteristics of the patients with fatigue

Variables	Spearman’s rank-order Correlation coefficient	*p*
Gender	0.119	0.297
Age, y	−0.223	0.049*
Onset age, y	−0.250	0.026*
Duration of disease	0.123	0.282
Education, y	0.010	0.929
LEDD	0.003	0.976
PFS total score	−0.155	0.173
H-Y stage	0.089	0.436
UPDRS total score	0.033	0.772
UPDRS part I	0.053	0.645
UPDRS part II	−0.029	0.798
UPDRS part III	0.074	0.515
UPDRS part IV	0.032	0.781
HAMD	−0.318	0.007*
PDSS	0.120	0.324
MoCA	0.123	0.280

*Significant difference. *H*-*Y* stage Hoehn & Yahr staging, *LEDD* levodopa equivalent daily dosage, *UPDRS* Unified Parkinson’ Disease Rating Scale, *MoCA* Montreal Cognitive Assessment, *HAMD* Hamilton Depression Scale score, *PDSS* Parkinson’s Disease Sleep Scale

To identify independent factors influencing dopaminergic responsive fatigue, variables significantly associated with fatigue remission rate in the bivariate correlation analyses (Table 4) were entered as independent variables into a logistic regression model. Only depression severity (odds ratio = 1.182, 95%CI 1.044–1.338, *P* = 0.008) was identified as an independent risk factor for dopaminergic drug non-responsive fatigue.

## Discussion

In the present study, fatigue problems were found in more than half of the PD patients (59.46 %) in northeastern China, which is consistent with previous reports [3, 22–24]. Moreover, fatigue appeared to be related with prolonged disease duration, increased disease severity, enhanced depressive symptoms, serious sleep disturbance, and the development of motor symptoms, except tremors. A univariate logistic regression analysis demonstrated that fatigue was significantly associated with several clinical characteristics of PD patients. Among the variables examined, only sleep disturbance was identified as an independent risk factor for fatigue. In addition, fatigue remission was found in 43.04 % of fatigued patients taking dopaminergic drugs. The development of DDNRF was associated with an older age and depressive symptoms, and depression severity was an independent risk factor for dopaminergic drug non-responsive fatigue. Our findings provide basic evidence for understanding the clinical significance of the non-motor symptom of fatigue in PD.

### Sleep disturbance is an independent risk factor for fatigue in PD patients

Sleep is necessary for the maintenance of mammalian homeostasis. Additionally, sleep is one of the most crucial determinants of fatigue severity [25]. In the general population, high-quality sleep is associated with less fatigue [26, 27]. Sleep disturbance has been associated with fatigue in several disorders, including cancer [28], multiple sclerosis [29] and traumatic brain injury [30]. In addition, it was reported that dissatisfaction with sleep, and not sleep itself, was associated with fatigue in psychotic patients [31].

Although the mechanisms of sleep abnormalities and fatigue have not yet been clarified, increased inflammatory cytokine release has been associated with poor sleep conditions and fatigue [32]. Specifically, the production of interleukin (IL)-1β and tumor necrosis factor (TNF)-α promotes non-rapid eye movement sleep (NREMS) under physiological and inflammatory conditions. Disturbed NREMS is associated with abnormal cyclic alternating pattern (CAP) rates and electroencephalogram arousals, and it is correlated with fatigue and sleepiness [33]. IL-1β and TNF-α alter neuronal excitability and induce symptoms associated with sleep loss by binding to their neural receptors, which subsequently regulate neurotransmitters or neuromodulators [34]. Moreover, the neurotransmitter serotonin governs sleep-wake behavior [35], and a reduction of serotonin transporters was found in subjects with fatigue syndrome [36]. These findings suggest that serotoninergic functions may play pivotal roles in controlling sleep and fatigue.

Fatigue, sleep disturbance and depression are the primary neuropsychiatric manifestations of PD, and they are related to patient quality of life [37, 38]. In this study, a logistic regression analysis revealed that sleep disturbance is the only independent factor for fatigue in PD patients, which is in accordance with the findings of a previous study [6]. Emerging lines of evidence indicate that sleep disorder treatment is effective for improving fatigue in patients with multiple sclerosis [39, 40]. Similarly, the management of sleep loss may provide a valuable approach for improving fatigue in PD patients. It has been proposed that fatigue stems at least in part from disturbed cytokine production in diseases such as PD [41]. Indeed, elevated secretion of IL-1β and TNF-α have been detected in the cerebrospinal fluid (CSF) of PD patients with sleep problems [42]. The circulating level of IL-1β is also increased in PD patients with fatigue [43]. Pavese et al. indicated that in addition to increased inflammatory cytokine production, fatigue in PD patients might result from serotonergic (5-hydroxytryptamine, 5-HT) dysfunction in the basal ganglia and limbic circuitry [44]. Considering the crucial roles that cytokine release and serotonergic functioning play in regulating sleep and fatigue, we hypothesize that sleep disturbance and fatigue may share a similar mechanism in the pathogenesis of PD, with abnormal cytokine release and serotonergic dysfunction being the primary components of this mechanism. Future studies should explore the molecular mechanisms involved in this process.

### Dopamine imbalance is associated with fatigue in PD patients

Another important finding of the current study was that dopaminergic medication improved fatigue in 43.04 % of PD patients with fatigue problems, whereas more than half of the fatigued patients were not responsive to dopaminergic medication. This result is consistent with ELLDOPA study [45], which showed that levodopa/carbidopa is effective in reducing the progression of fatigue in drug naïve PD patients [12]. Moreover, the study of ADAGIO [46] also showed that rasagiline was associated with significantly less progression of fatigue compared with placebo over a 9-month period in drug naïve patients with PD [47]. Both carbidopa/levodopa and rasagiline are dopaminergic drugs, which increase the extracellular levels of dopamine. Thus, we infer dopamine dysfunction may involve in the pathogenesis of fatigue in PD. The effects we observed might be due to enhanced dopaminergic neurotransmission, indicating the therapeutic value of dopaminergic treatment for fatigue relief in PD. However, according to the univariate logistic regression and correlation analysis performed in our study, there are no significant associations among fatigue, fatigue severity, remission rate, and dopaminergic medication dosage because a no dose-effect correlation was detected between levodopa and fatigue. Hence, levodopa may play a role in the mechanism underlying the development of fatigue, which is consistent with other current results. The dopamine imbalance hypothesis of fatigue was recently presented in multiple sclerosis and other neurological disorders [48]. Thus, restoring dopamine levels in the CNS by means of dopaminergic medication, such as levodopa, might be an essential strategy for the treatment of fatigue in PD.

### Depression is an independent risk factor for dopaminergic drug non-responsive fatigue

Depression is another prominent non-motor symptom in PD [49]. Fatigue in PD is often associated with depression, using a logistic regression model, we further demonstrated that depression was the only independent factor for the efficacy of dopaminergic medication in treating fatigue in PD patients, which might imply crosstalk between depression, fatigue and dopamine insufficiency.

Pavese et al. found that compared with patients without fatigue, PD patients with fatigue showed greatly reduced serotonin transporter binding in the basal ganglia and limbic circuitry, as well as a significant reduction in dopamine uptake in the caudate and insula [44]. These results demonstrate that even though a dysfunctional serotonergic pathway plays a predominant role in fatigue, reduced dopaminergic functioning also contributes to the incidence of fatigue [44]. Hence, both dopaminergic and serotonergic pathways contribute to fatigue in PD patients. Interestingly, other studies have indicated that excessive 5-HT also induced fatigue. Indeed, increased 5-HT and decreased dopamine concentrations were detected in the brain in a rat model of exercise-induced fatigue [50]. These findings imply that the level of 5-HT may not be a determinant, and maintaining the balance between serotonergic and dopaminergic function is key for fatigue relief. Hence, dopamine administration may improve fatigue only in PD patients who have a functional serotonergic system with normal 5-HT concentrations and receptor functioning, which is consistent with our results. In other words, functional serotonergic pathway is necessary for dopamine to alleviate fatigue. Therefore, both 5-HT and dopamine should be considered during the treatment of PD fatigue. For instance, in the early stage of PD, a supplement of only 5-HT without levodopa medication may be insufficient for relieving fatigue, as levodopa is usually not prescribed early in PD due to the absence of obvious motor symptoms. Similarly, when fatigue is present with no obvious depression, a combined administration of levodopa with 5-HT, but not levodopa alone, may provide greater relief from fatigue. Thus, we hypothesize that dopaminergic medication is required, but not sufficient, for fatigue suppression in fatigued PD patients with moderate depression, and such treatment can restore serotonergic neurotransmission and serve as a combination therapy. This may offer an ideal strategy for the treatment of fatigue in PD patients.

## Conclusions

In the present study, we found that fatigue is common in PD patients. PD patients with severe sleep disturbances tend to suffer from fatigue. Levodopa improved fatigue only in PD patients with mild depression or no depression, restoring serotonergic neurotransmission as a combination therapy may offer a better strategy for the treatment of fatigue in these patients.

### Consent

Written informed consent was obtained from the patient for the publication of this report and any accompanying images.
